# Supporting Adolescents Bereaved by Sibling Suicide: A Review of Needs, Gaps, and Opportunities

**DOI:** 10.1007/s40124-025-00363-9

**Published:** 2025-09-24

**Authors:** Sarah Danzo, Yomna Anan, Kacie Kidd, Gina Sequeira, Nicole F. Kahn

**Affiliations:** 1 Department of Psychiatry and Behavioral Sciences, University of Washington School of Medicine, Seattle, WA USA; 2 Center for Child Health, Behavior & Development, Seattle Children’s Research Institute, Seattle, WA USA; 3 Department of Pediatrics, West Virginia University School of Medicine, Morgantown, WV USA; 4 Department of Pediatrics, University of Washington School of Medicine, Seattle, WA USA

**Keywords:** Review, Adolescents, Sibling, Bereavement, Suicide, Prevention

## Abstract

**Purpose of Review:**

Suicide is a leading cause of death among adolescents. However, while there is a growing focus on suicide prevention efforts, there has been very little research on how to support young people bereaved by suicide, particularly adolescent siblings. Given the impact of suicide on the entire family, as well as the increased risk of mental health problems and suicidal behaviors during adolescence, we sought to review the current literature through a family systems lens to identify what is known, current gaps, and needs for future work to be able to better support adolescents bereaved by sibling suicide.

**Recent Findings:**

Overall, very few studies have examined the impact of sibling suicide on adolescents, and even fewer examine interventions for adolescents who have lost a sibling to suicide.

**Summary:**

Findings highlight a critical need for more work focused on developing and rigorously evaluating interventions for adolescents bereaved by sibling suicide. Additional program development and research in this area is critical to improve long term outcomes for adolescents and their families, and to ultimately reduce suicide risk among surviving adolescent siblings.

## Introduction

Adolescence represents a developmental period in which individuals are at heightened risk for emotional and behavioral problems, including depression and suicidal thoughts and behaviors (STB) [1]. Indeed, suicide is a major public health concern among adolescents in the United States [2, 3]. In 2023, 20.4% of U.S. high school students reported having seriously considered attempting suicide and 9.5% of high schoolers reported making a suicide attempt within the past year [4]. In line with this, suicide is currently a leading cause of death among U.S. adolescents and young adults (AYA) ages 10–34 years old [3, 5]. However, while there has been growing attention to the youth mental health crisis and efforts to reduce death by suicide, there has been less attention on how to support the individuals left behind after suicide [6, 7].

Notably, research demonstrates that in addition to adolescence being a developmental period in which individuals are at heightened risk for suicide, adolescents are also particularly negatively impacted by exposure to others’ STB [1, 8–10]. For example, knowledge about peers’ STB can increase adolescent’s own suicide risk, and adolescents who know a peer who engages in self-harm or STB are themselves more likely to also self-harm or have STB [10, 11]. Additionally, adolescents bereaved by suicide are at increased risk of experiencing complicated grief reactions and engaging in suicidal behaviors themselves compared to adolescents bereaved by non-suicide deaths [8, 11–14].

Despite the impact of suicide bereavement, there is limited research investigating the impact of a sibling’s suicide on surviving adolescent siblings [15]. Instead, research typically focuses on the impact of losing a child to suicide on surviving parents [16, 17]. However, sibling relationships are some of the most influential and longest-lasting relationships in a person’s life, and death of a sibling impacts surviving adolescents both directly, as well as indirectly through the impact on the larger family system [15]. While limited, research highlights that experiencing the death of a sibling is often associated with more negative outcomes compared to experiencing the death of someone from a different type of relationship [18, 19]. Further, Tidemalm and colleagues (2011) found that individuals who share the same home environment as a sibling who died by suicide have the highest risk of suicide compared to all other types of family members [19]. Another study that examined the impact of types of loss (e.g., sibling or close friend), regardless of cause of death, on young adult outcomes found that participants who lost a sibling were significantly more likely to experience complicated grief and have higher levels of depression, grief, and somatic symptoms compared to those who lost a friend [18].

Studies have also shown that having a sibling die by suicide during adolescence significantly increases the risk of developing emotional and behavioral problems. For example, adolescents have a seven times greater risk of experiencing major depression within the six months following the death of a sibling when the cause of death is suicide compared to any other cause of death [20]. Adolescents bereaved by sibling suicide have also been shown to experience significant symptoms of complicated and prologued grief reactions, trauma, academic challenges, feelings of guilt, and increased STB [8, 18, 19, 21, 22].

Given both the rising rates of suicide among AYA and the known impact of suicide exposure on adolescents, it is critical that pediatric care providers understand the impact of suicide bereavement on surviving siblings and implement appropriate interventions. Improved understanding in this area will promote the development of more accessible and effective intervention programs to support these youth and reduce subsequent mental health problems and suicide risk. Thus, the purpose of this review is to highlight (1) existing prevention and intervention strategies to support surviving siblings, (2) gaps in the literature, and (3) promising areas for future prevention and intervention development to support adolescents who have lost a sibling to suicide.

### Theoretical Framework

Family Systems Theory (FST), first described by Bowen in 1966, represents the family as an interconnected unit such that influences on any member of the family have ramifications for all members [23]. While initially based on observations of families experiencing schizophrenia, FST has expanded to further understanding how multigenerational family relationships and patterns can impact the development and/or persistence of other psychological disorders such as depression and anxiety [24, 25]. FST thus serves as a basis for modern family-based therapeutic approaches and aids in our understanding of the likely impacts of suicide on siblings.

In line with FST, it is important to consider how adolescent suicide affects parents/caregivers (hereafter referred to as parents), as their experiences are inextricably linked with those of their surviving children. Losing a child to suicide is associated with complicated grief, as well as enduring and significantly impairing psychological symptoms including increased distress, anxiety, depression, feelings of guilt, and STB. Parents bereaved by suicide also report increased conflict with other family members, friends, and co-workers following the death of their loved one, leading to increased relational problems and avoidance of social situations [26]. Consequently, parents are often in obvious need of support following the death of a child. In response to this, most family and community support efforts after a child’s suicide tends to focus on providing emotional, instrumental, and informational support to bereaved parents. Such support often includes providing empathy or comfort, providing practical assistance, such as helping with childcare or finances, or providing advice, knowledge, or resources to parents. However, while youth might benefit from similar types of support, surviving siblings often do not receive the same attention as surviving parents, resulting in surviving siblings often becoming the “forgotten bereaved” [15, 21].

Notably, while siblings are frequently overlooked after adolescent suicide, many also take on new roles within the family. This is due to several reasons. First, adolescents may avoid expressing their feelings of sadness, anger, guilt, or shame following the death of their sibling so as not to further upset their parents [27]. They may also be expected to put the needs of other family members, such as parents and younger siblings, before their own as they grieve. This can in turn mean that youth take on more caretaking roles as their parents cope with the death of their child [15, 21, 27]. These experiences can contribute to further negative mood symptoms and withdrawal from both family members and from peers and other support systems. Together, this leads to a multitude of negative outcomes for surviving siblings [21, 28, 29] and suggests that while there is likely some overlap in the kind of support adolescents bereaved by sibling suicide need compared to parents, they likely have additional needs compared to parents as well. For these reasons, support and interventions for surviving adolescent siblings may need to look slightly different than interventions focused on their caregivers.

### Existing Postventions for Family Members Bereaved by Suicide

In general, individuals bereaved by suicide tend to receive less bereavement support than those who have lost a loved one due to other causes of sudden death [30]. Consequently, most individuals who have lost someone to suicide report wanting more support [30]. While there are some formal and informal interventions for family members after the loss of a child to suicide, these interventions have limited evidence of effectiveness and very few are tailored to adolescent siblings [31].

Broadly, studies examining suicide bereavement interventions have reported on a wide range of interventions and participant types, with various key components and implementation strategies, as well as timing of intervention delivery following bereavement, ranging from a few days to a few years [32, 33]. These interventions include individual, family, and group based interventions, as well as interventions that utilize a range of psychoeducation, therapeutic techniques, and support services [34]. However, most studies discuss processes, theory, or implementation of these interventions, with a noticeable lack of detailed program content or rigorous evaluation of effectiveness. As a result, systematic reviews and meta-analyses examining the effectiveness of interventions for people who have lost someone to suicide highlight several methodological challenges and limitations to assessing the effectiveness of the limited number of suicide bereavement interventions that exist [34, 35].

To date, studies evaluating bereavement interventions specifically for youth have largely focused on children who have lost a parent or any relative to suicide, rather than a sibling [36, 37]. Unfortunately, this published literature also lacks detail on the intervention content. Regarding the effectiveness of suicide bereavement interventions for youth, findings are mixed. One study demonstrated positive outcomes, including reductions in anxiety and depression, for youth who participated in a 10-session group bereavement intervention focused on psychoeducation and problem solving following the loss of a family member to suicide [37]. However, Currier and colleagues (2007) conducted a meta-analysis to assess the effectiveness of bereavement interventions (not specific to suicide) with youth and found that overall, evidence did not support that group bereavement interventions had a significant impact on youth adjustment or overall functioning. However, they did note that intervention delivery closer to time of death appeared to increase intervention effectiveness [38].

Although more research with youth is clearly needed, the existing literature highlights *promising* program components that could be tailored to support adolescents bereaved by sibling suicide. For example, research with adults bereaved by spousal suicide found that adults who participated in an 8-week group bereavement or social group postvention program reported reduced anxiety, depression, distress, and improved social adjustment following intervention completion [39, 40]. Although these studies suggest promise for group interventions, they unfortunately lack details on what specifically these group interventions entailed. Importantly, results from meta-analyses and individual studies suggest that it may be that not all individuals benefit from bereavement interventions. Instead, it appears that individuals who experience more maladjustment and psychological distress are the most likely to benefit and should be prioritized for these interventions [38, 41].

Despite this, there is currently insufficient information available to determine if, and what components of, bereavement interventions have a significant impact. A recent systematic review showed that across studies that examined various grief interventions including those based on supportive psychotherapy, cognitive behavioral therapy (CBT), and family grief counseling for people bereaved by suicide across the lifespan, most demonstrated non-significant improvements, indicating a need for additional research in this area with larger sample sizes [34]. Thus, more research is needed to extend existing research to youth bereaved by suicide, as well as to identify when and for whom these interventions would have the most positive impacts.

### Synthesis: so What?

#### Future Research Considerations

 There are several gaps in this area that future work needs to address. First, the literature on the psychosocial functioning and bereavement experiences of adolescents who have lost a sibling to suicide is significantly lacking [42, 43]. The majority of the research in this area either occurred in the 1990 s and early 2000 s [20, 21] or has focused primarily on the experiences of adults [44, 45]. Similarly, there is limited research on the effectiveness of interventions/postventions for youth who have lost a sibling to suicide. Specifically, few studies include a description of the interventions used or rigorous evaluations (i.e., randomization, longitudinal follow-up, sufficient sample sizes), and most have an overrepresentation of female and adult participants, even if those participants were an adolescent when they lost their sibling to suicide. To our knowledge, only one such study has been conducted with youth who have lost a sibling to suicide [37] and none focus explicitly on the experiences of adolescents versus other family members. Thus, further research and program development are desperately needed to understand and address the unique developmental experiences and needs of adolescents who have lost a sibling to suicide.

Ultimately, while more work is needed to better understand the impacts of adolescent suicide on surviving siblings, it is also critical that learned information be translated into interventions to support surviving adolescents. Future research should apply a family systems lens in order to take into account the complex processes within the family following death by suicide of a child [23]. For example, existing studies highlight that while caregivers are significantly negatively impacted when a child dies by suicide [26], surviving siblings that still live at home, and especially younger siblings, are often the most negatively impacted when an adolescent sibling dies by suicide [20, 21, 46]. Thus, there is a need to better understand the independent impacts that losing a sibling to suicide has on surviving adolescents, how parents and the family system are impacted, and any interaction or trickle down effects these may have on surviving children [15].

#### **Intervention and Program Development Considerations**

There is also a critical need to develop and evaluate interventions for youth bereaved by sibling suicide to ameliorate potential long-term negative impacts. Development of such interventions should consider individual’s unique reactions to grief as well as complicated grief reactions, such as prolonged acute grief that interferes with daily functioning and grief that adolescents have difficulty voicing when trying to protect surviving parents. Additionally, interventions should assess and account for fluctuating family system dynamics including changes in relationships and interactions between surviving family members [14, 21]. In line with this, Brent and colleagues (1993, 1996) proposed the need for family-based approaches to support both surviving siblings and caregivers bereaved by a child suicide [20, 46]. While this is likely an important avenue, development of independent interventions for surviving siblings, separate from their caregivers, should also be considered.

Specific intervention components should also be assessed when developing these interventions. For example, components such as the timing of intervention delivery in relation to suicide bereavement, accessibility of interventions, and feasibility of adolescents being able to independently enroll and/or engage in the intervention with limited parental support, are all important to consider. Additionally, programs should consider tailoring intervention content and support to match the needs of surviving siblings and caregivers through both an individual and holistic lens to address individual needs as well as address the context in which they are living [47]. For example, it may be important to consider the nature of suicide to determine if method of suicide or medical treatment duration prior to death impacts surviving adolescent outcomes. Similarly, it may be important to assess individual differences, such as if the surviving adolescent knew their sibling had been struggling with STB, and how they were exposed to their sibling’s STB in order to provide more targeted support.

Future research can also apply a preventative lens to such interventions to reduce risk of the development of mental health problems and STB. For example, it may be that adolescents who have a sibling engaged in STB should also be offered preventative supportive interventions to provide support, validation, and coping skills. If death by suicide does occur, we must also determine the point following death – immediately following, a few weeks after, or a few months after – at which tertiary prevention interventions should be delivered to optimize outcomes and overall functioning [48]. Finally, and perhaps most importantly, future research aimed at developing such interventions would benefit from using user-centered design approaches and co-design methods that involve people with lived experience of losing a child or sibling to suicide in order to ensure that these interventions are tailored to the unique needs of these populations [47].

## Conclusion

Sibling relationships are often one of the most influential relationships in a person’s life [15]. However, little research has focused on how to best support adolescents following the suicide of a sibling. While the impact of losing a sibling to suicide is still not well understood, research to date highlights that losing a sibling to suicide is associated with several negative outcomes including increased grief, trauma, academic challenges, depression, and STB [8, 18, 19, 22]. Despite these negative outcomes, there has been essentially no progress over the past 20 years in developing and evaluating interventions to support adolescents bereaved by sibling suicide. Thus, there is a critical need for a re-initiation of research in this area to better understand both the short and long-term impacts of sibling suicide bereavement on adolescents, as well as to develop, describe, and rigorously evaluate interventions to improve the overall well-being and long-term outcomes for this too often overlooked population.

## Key references

- Royden LJ. Forgotten but not gone: a heuristic literature review of sibling suicidebereavement. Aotearoa N Z Soc Work. 2021;33(2):19–31.
  - A recent literature review of how suicide affects siblings.
- Graham F, Bartik W, Wayland S, Maple M. Effectiveness and acceptability ofinterventions offered for those bereaved by parental loss to suicide in childhood: amixed methods systematic review. Arch Suicide Res Off J Int Acad Suicide Res.2025;29(1):45–76.
  - Recent review of studies assessing interventions for youth bereavement of a parent.

## Data Availability

No datasets were generated or analysed during the current study.

## References

[1] Lin J, Guo W. The research on risk factors for adolescents’ mental health. Behav Sci (Basel). 2024;14(4):263. 38667059 10.3390/bs14040263 PMC11047495

[2] CDC. Injury Center. 2025 [cited 2025 Aug 17]. Data, Statistics, and Reporting. Available from: https://www.cdc.gov/injury/data-research/index.html

[3] Centers for Disease Control and Prevention [Internet]. [cited 2025 Aug 17]. CDC WISQARS - Web-based Injury Statistics Query and Reporting System. Available from: https://wisqars.cdc.gov/

[4] Verlenden JV, Mental Health. and Suicide Risk Among High School Students and Protective Factors — Youth Risk Behavior Survey, United States, 2023. MMWR Suppl [Internet]. 2024 [cited 2025 May 18];73. Available from: https://www.cdc.gov/mmwr/volumes/73/su/su7304a9.htm 10.15585/mmwr.su7304a9 PMC11559681 39378246

[5] CDC WONDER [Internet]. [cited 2025 Aug 17]. Available from: https://wonder.cdc.gov/

[6] 2024 National Strategy for Suicide Prevention. 2024.

[7] CDC. Youth Risk Behavior Surveillance System (YRBSS). 2025 [cited 2025 Aug 17]. YRBS Data Summary & Trends Report. Available from: https://www.cdc.gov/yrbs/dstr/index.html

[8] Del Carpio L, Paul S, Paterson A, Rasmussen S. A systematic review of controlled studies of suicidal and self-harming behaviours in adolescents following bereavement by suicide. PLoS ONE. 2021;16(7):e0254203. 34242305 10.1371/journal.pone.0254203 PMC8270178

[9] Hawton K, Hill NTM, Gould M, John A, Lascelles K, Robinson J. Clustering of suicides in children and adolescents. Lancet Child Adolesc Health. 2020;4(1):58–67. 31606323 10.1016/S2352-4642(19)30335-9

[10] Reyes-Portillo JA, Lake AM, Kleinman M, Gould MS. The relation between descriptive norms, suicide ideation, and suicide attempts among adolescents. Suicide Life Threat Behav. 2019;49(2):535–46. 29470851 10.1111/sltb.12446 PMC6105573

[11] Quigley J, Rasmussen S, McAlaney J. The associations between children’s and adolescents’ suicidal and self-harming behaviors, and related behaviors within their social networks: a systematic review. Arch Suicide Res Off J Int Acad Suicide Res. 2017;21(2):185–236. 10.1080/13811118.2016.1193075 27267251

[12] Jordan JR. Bereavement after Suicide. Psychiatr Ann [Internet]. 2008 [cited 2025 Aug 18];38(10). Available from: https://journals.healio.com/doi/10.3928/00485713-20081001-05

[13] Shear MK, Simon N, Wall M, Zisook S, Neimeyer R, Duan N, et al. Complicated grief and related bereavement issues for DSM-5. Depress Anxiety. 2011;28(2):103–17. 21284063 10.1002/da.20780 PMC3075805

[14] Tal Young I, Iglewicz A, Glorioso D, Lanouette N, Seay K, Ilapakurti M, et al. Suicide bereavement and complicated grief. Dialogues Clin Neurosci. 2012;14(2):177–86. 22754290 10.31887/DCNS.2012.14.2/iyoung PMC3384446

[15] Royden LJ. Forgotten but not gone: a heuristic literature review of sibling suicide bereavement. Aotearoa N Z Soc Work. 2021;33(2):19–31.

[16] Ratnarajah D, Schofield MJ. Parental suicide and its aftermath: a review. J Fam Stud. 2007;13(1):78–93.

[17] Schreiber JK, Sands DC, Jordan JR. The perceived experience of children bereaved by parental suicide. Omega. 2017;75(2):184–206. 28490278 10.1177/0030222815612297

[18] Herberman Mash HB, Fullerton CS, Ursano RJ. Complicated grief and bereavement in young adults following close friend and sibling loss. Depress Anxiety. 2013;30(12):1202–10. 23401012 10.1002/da.22068

[19] Tidemalm D, Runeson B, Waern M, Frisell T, Carlström E, Lichtenstein P, et al. Familial clustering of suicide risk: a total population study of 11.4 million individuals. Psychol Med. 2011;41(12):2527–34. 21733212 10.1017/S0033291711000833 PMC3207221

[20] Brent DA, Perper JA, Moritz G, Liotus L, Schweers J, Roth C, et al. Psychiatric impact of the loss of an adolescent sibling to suicide. J Affect Disord. 1993;28(4):249–56. 8227761 10.1016/0165-0327(93)90060-w

[21] Dyregrov K, Dyregrov A. Siblings after suicide–the forgotten bereaved. Suicide Life Threat Behav. 2005;35(6):714–24. 16552987 10.1521/suli.2005.35.6.714

[22] Rostila M, Berg L, Saarela J, Kawachi I, Hjern A. Experience of sibling death in childhood and risk of psychiatric care in adulthood: a national cohort study from Sweden. Eur Child Adolesc Psychiatry. 2019;28(12):1581–8. 30937545 10.1007/s00787-019-01324-6 PMC6861357

[23] Bowen M. The use of family theory in clinical practice. Compr Psychiatry. 1966;7(5):345–74. 5922263 10.1016/s0010-440x(66)80065-2

[24] Cicchetti D, Toth SL. Developmental perspectives on depression. University Rochester; 1992. p. 428.

[25] Besharat MA, Rostami R, Gharehbaghy F, Gholamali Lavasani M. Differentiation and chronic anxiety symptoms in family emotional system: designing and evaluating the effectiveness of an intervention based on Bowen family systems theory. Fam Couns Psychother. 2015;5(1):1–24.

[26] Ross V, Kõlves K, Kunde L, De Leo D. Parents’ experiences of suicide-bereavement: a qualitative study at 6 and 12 months after loss. Int J Environ Res Public Health. 2018;15(4):618. 29597297 10.3390/ijerph15040618 PMC5923660

[27] Shields C, Kavanagh M, Russo K. A qualitative systematic review of the bereavement process following suicide. OMEGA - J Death Dying. 2017;74(4):426–54. 10.1177/0030222815612281 28355992

[28] Adams E, Hawgood J, Bundock A, Kõlves K. A phenomenological study of siblings bereaved by suicide: a shared experience. Death Stud. 2019;43(5):324–32. 29757098 10.1080/07481187.2018.1469055

[29] Terkamo-Moisio A, Järvi J, Aho AL. The life changes of sibling survivors after suicide – A qualitative study. Mortality. 2025;0(0):1–15.

[30] Pitman A, Osborn D, King M, Erlangsen A. Effects of suicide bereavement on mental health and suicide risk. Lancet Psychiatry. 2014;1(1):86–94. 26360405 10.1016/S2215-0366(14)70224-X

[31] Journot-Reverbel K, Raynaud JP, Bui E, Revet A. Support groups for children and adolescents bereaved by suicide: lots of interventions, little evidence. Psychiatry Res. 2017;250:253–5. 28171792 10.1016/j.psychres.2017.01.077

[32] Hazell P, Lewin T. An evaluation of postvention following adolescent suicide. Suicide Life Threat Behav. 1993;23(2):101–9. 8342209

[33] Zisook S, Shear MK, Reynolds CF, Simon NM, Mauro C, Skritskaya NA, et al. Treatment of complicated grief in survivors of suicide loss: a HEAL report. J Clin Psychiatry. 2018;79(2):17m11592. 29617064 10.4088/JCP.17m11592

[34] Andriessen K, Krysinska K, Hill NTM, Reifels L, Robinson J, Reavley N, et al. Effectiveness of interventions for people bereaved through suicide: a systematic review of controlled studies of grief, psychosocial and suicide-related outcomes. BMC Psychiatry. 2019;19(1):49. 30700267 10.1186/s12888-019-2020-z PMC6354344

[35] McDaid C, Trowman R, Golder S, Hawton K, Sowden A. Interventions for people bereaved through suicide: systematic review. Br J Psychiatry. 2008;193(6):438–43. 19043143 10.1192/bjp.bp.107.040824

[36] Graham F, Bartik W, Wayland S, Maple M. Effectiveness and acceptability of interventions offered for those bereaved by parental loss to suicide in childhood: a mixed methods systematic review. Arch Suicide Res Off J Int Acad Suicide Res. 2025;29(1):45–76. 10.1080/13811118.2024.2351101 38767988

[37] Pfeffer CR, Jiang H, Kakuma T, Hwang J, Metsch M. Group intervention for children bereaved by the suicide of a relative. J Am Acad Child Adolesc Psychiatry. 2002;41(5):505–13. 12014782 10.1097/00004583-200205000-00007

[38] Currier JM, Holland JM, Neimeyer RA. The effectiveness of bereavement interventions with children: a meta-analytic review of controlled outcome research. J Clin Child Adolesc Psychol Off J Soc Clin Child Adolesc Psychol Am Psychol Assoc Div 53. 2007;36(2):253–9. 10.1080/15374410701279669 17484697

[39] Constantino RE, Bricker PL. Nursing postvention for spousal survivors of suicide. Issues Ment Health Nurs. 1996;17(2):131–52. 8707534 10.3109/01612849609035002

[40] Constantino RE, Sekula LK, Rubinstein EN. Group intervention for widowed survivors of suicide. Suicide Life Threat Behav. 2001;31(4):428–41. 11775718 10.1521/suli.31.4.428.22044

[41] Andriessen K, Krysinska K. Essential questions on suicide bereavement and postvention. Int J Environ Res Public Health. 2012;9(1):24–32. 22470275 10.3390/ijerph9010024 PMC3315078

[42] Andriessen K, Rahman B, Draper B, Dudley M, Mitchell PB. Prevalence of exposure to suicide: a meta-analysis of population-based studies. J Psychiatr Res. 2017;88:113–20. 28199930 10.1016/j.jpsychires.2017.01.017

[43] Huff MB. Effects of youth suicide upon siblings left behind: an untold story. In Omar HA, editor. Youth suicide prevention: Everybody’s business. New York: Novinka/Nova Science; 2015. pp. 63–72.

[44] Powell KA, Matthys A. Effects of suicide on siblings: uncertainty and the grief process. J Fam Commun. 2013;13(4):321–39.

[45] Blaze P, Roberts RM. Support after suicide: A thematic analysis of siblings’ experience. OMEGA - J Death Dying. 2023;0(0):1-19. 10.1177/00302228231195922. 10.1177/00302228231195922 PMC12769914 37574903

[46] Brent DA, Moritz G, Bridge J, Perper J, Canobbio R. The impact of adolescent suicide on siblings and parents: a longitudinal follow-up. Suicide Life Threat Behav. 1996;26(3):253–9. 8897664

[47] Ross AM, Krysinska K, Rickwood D, Pirkis J, Andriessen K. How best to provide help to bereaved adolescents: a Delphi consensus study. BMC Psychiatry. 2021;21(1):1–10. 34814884 10.1186/s12888-021-03591-7 PMC8609510

[48] Kabatchnick R, Black BP. Remembering the forgotten bereaved: Understanding and caring for siblings of completed suicide victims. In Black BP, Wright PM, Limbo R, editors. Perinatal and pediatric bereavement in nursing and other health professions. New York: Springer Publishing Company; 2016. pp. 287–305.

